# Development and validation of a cytochrome *c*-coupled assay for pteridine reductase 1 and dihydrofolate reductase

**DOI:** 10.1016/j.ab.2009.09.003

**Published:** 2010-01-15

**Authors:** Emma J. Shanks, Han B. Ong, David A. Robinson, Stephen Thompson, Natasha Sienkiewicz, Alan H. Fairlamb, Julie A. Frearson

**Affiliations:** Division of Biological Chemistry and Drug Discovery, College of Life Sciences, University of Dundee, Dundee DD1 5EH, UK

**Keywords:** Drug discovery, Screening, Pteridine reductase, Dihydrofolate reductase

## Abstract

Activity of the pterin- and folate-salvaging enzymes pteridine reductase 1 (PTR1) and dihydrofolate reductase–thymidylate synthetase (DHFR-TS) is commonly measured as a decrease in absorbance at 340 nm, corresponding to oxidation of nicotinamide adenine dinucleotide phosphate (NADPH). Although this assay has been adequate to study the biology of these enzymes, it is not amenable to support any degree of routine inhibitor assessment because its restricted linearity is incompatible with enhanced throughput microtiter plate screening. In this article, we report the development and validation of a nonenzymatically coupled screening assay in which the product of the enzymatic reaction reduces cytochrome *c,* causing an increase in absorbance at 550 nm. We demonstrate this assay to be robust and accurate, and we describe its utility in supporting a structure-based design, small-molecule inhibitor campaign against *Trypanosoma brucei* PTR1 and DHFR-TS.

Human African trypanosomiasis (HAT), 1 also known as sleeping sickness, is a protozoan parasitic disease that causes death and disability across large undeveloped regions of sub-Saharan Africa [1]. Infection is caused by two subspecies of *Trypanosoma brucei*: *T. b. gambiense* (endemic in central and western areas) and *T. b. rhodesiense* (predominantly found in southern and eastern Africa). Both infections are fatal if left untreated. The early (hemolymphatic) stage of the disease is treated by suramin or pentamidine, and the late (central nervous system) stage is treated by eflornithine (*T. b. gambiense*) or the arsenical drug melarsoprol (both forms). All currently available drugs must be given by injection and suffer from serious deficiencies such as unacceptable toxicity, poor efficacy, high cost, and the need for prolonged hospitalization.

Development of drug therapies for HAT has been largely disregarded over the past 60 years. Investment in drug discovery for this and other “orphan” diseases has declined sufficiently such that less than 1% of all new drugs over the past 25 years have targeted tropical parasitic diseases [2]. This is largely due to the poverty associated with these neglected diseases and the consequent low profitability of developing and manufacturing drugs.

Pterins and folates are known to be essential for growth in *Leishmania* spp. and related trypanosomatids [3,4], and biosynthetic enzymes for de novo synthesis are lacking from their respective genomes [5]. Consequently, trypanosomatids possess multiple biopterin and folate transporters with salvage pathways to convert them into the reduced cofactors 5,6,7,8-tetrahydrobiopterin (H_4_B) and 5,6,7,8-tetrahydrofolate (H_4_F) by means of pteridine reductase 1 (PTR1, EC 1.5.1.33) and the bifunctional, nicotinamide adenine dinucleotide phosphate (NADPH)-dependent enzyme dihydrofolate reductase–thymidylate synthase (DHFR-TS, ECs 1.5.1.4 and 2.1.1.45, respectively). Due to the auxotrophic nature of these parasites for folates and pterins, the enzymes associated with their metabolism have been investigated extensively as potential drug targets [3,4].

DHFR-TS, together with serine hydroxymethyltransferase (SHMT) or the glycine cleavage system (the former enzyme is present in *Leishmania major* but not in *T. brucei* [5,6]), is responsible for de novo synthesis of thymidylate (dTMP) required for DNA replication. Gene knockout studies indicate that *dhfr-ts* null mutants of *L. major* and *T. brucei* are able to grow normally in culture provided that thymidine is present in the medium [7,8]. *T. brucei* DHFR-TS null mutants are unable to establish an infection in mice due to the extremely low concentrations of thymidine in plasma [8]. In contrast, *L. major* DHFR-TS null mutants cause low-grade infections in mice [9], presumably due to limited availability of thymidine in the infected macrophage.

Disappointingly, DHFR-TS inhibitors, such as those commonly used in the development of anticancer, antimalarial, and antibacterial drugs, have not shown equivalent efficacy against *T. brucei* [8] *.* As demonstrated elegantly by Beverley and coworkers for *L. major* [10,11], PTR1, a broad-specificity, short-chain dehydrogenase, can reduce dihydrofolate (H_2_F) to H_4_F, thereby circumventing inhibition of DHFR-TS. Therefore, it is likely that cooperative inhibition of both trypanosomal enzymes may be required to produce an effective therapy.

It is also possible that PTR1 may be a therapeutic target in its own right given that its primary function is to sequentially reduce biopterin (B) to 7,8-dihydrobiopterin (H_2_B) and H_4_B. The biological function of pterins in trypanosomatids is unknown, and many functions reported for mammalian cells have been discounted [3,12,13]. *L. major* *ptr1* ^−^ mutants are viable provided that the medium is supplemented with H_2_B and H_4_B [10,14]. However, similar gene knockout and knockdown studies on bloodstream *T. brucei* suggest that PTR1 is essential (N. Sienkiewicz and A. H. Fairlamb, unpublished).

The Drug Discovery Unit at the University of Dundee has been established to undertake a fully integrated approach to neglected disease drug discovery. Combining a variety of approaches to hit discovery, including high-throughput screening, ligand- and structure-based design with medicinal chemistry, and drug metabolism and pharmacokinetics (DMPK) capabilities, our goal is to deliver drug candidates for HAT for entry into formal preclinical development. To support this endeavor, a portfolio of targets has been compiled and assessed [15] at Dundee from worldwide research efforts, with *T. brucei* DHFR-TS (*Tb*DHFR-TS) and *T. brucei* PTR1 (*Tb*PTR1) ranking high on this prioritized list.

The assay principally used for measuring DHFR-TS and PTR1 activity follows a spectrophotometric decrease in absorbance at 340 nm, corresponding to oxidation of NADPH in the presence of H_2_F and H_2_B (or biopterin), respectively. However, this cuvette-based assay is unsuitable for supporting any level of throughput with the necessary robustness required to support a progressive medicinal chemistry effort. Similarly, detection of enzyme activity by high-performance liquid chromatography (HPLC) also presents insufficient throughput.

The development of a screening assay to support in vitro target activity assessment that provides relevant accurate data and performs consistently throughout a program contributes significantly to the success of a lead development project. Therefore, a robust “fit for purpose” assay is a necessity in discovering initial chemical starting points and the subsequent development of meaningful structure–activity relationships for compound series. To enable routine screening for small-molecule inhibitors of *Tb*DHFR-TS and *Tb*PTR1, we developed and validated a nonenzymatically coupled absorbance assay. Here the enzymatic products H_4_B and H_4_F are coupled to the reduction of oxidized cytochrome *c* (cyt *c* Fe^3+^), the formation of which can be measured as an increase in absorbance at 550 nm. The assay is executed in a 96-well microtiter plate, with performance statistics commensurate with the long-term support of a medicinal chemistry program.

## Materials and methods

### Cloning and protein production of *Tb*PTR1 and *Tb*DHFR-TS

All plasmids used were sequenced by the University of Dundee DNA Sequencing Service (http://www.dnaseq.co.uk) and verified against the published database sequences.

*Tb*PTR1 was expressed and purified as the N-terminal hexahistidine-tagged protein as described previously [16].

The 1584-nucleotide sequence (accession no. TBU20781) encoding the full-length *Tb*DHFR-TS was obtained by polymerase chain reaction (PCR) from *T. brucei* strain 427 genomic DNA (kindly provided by M. Lucia L. Güther, University of Dundee) using the forward primer 5′-CTGGATCCATGCTCAGTCTTACGCGTATCCTCCG-3′ and the reverse primer 5′-GACTCGAGCTACACCGCCATCTCCATAGAAATTACG-3′. The underlined bases represent restriction sites for *Bam*HI and *Xho*I, respectively, and were used for cloning into a pFastBac1 bacculovirus expression vector (Invitrogen) that had been modified to encode a glutathione *S*-transferase (GST) tag followed by a PreScission protease recognition sequence, resulting in the final bacculovirus expression construct pFastBac GST–*Tb*DHFR-TS. Recombinant protein was produced using the Bac-to-Bac system (Invitrogen) following the manufacturer’s protocol. Bacculovirus at a multiplicity of infection of 5 was used to infect *Spodoptera frugiperda* 21 cells (1.5 × 10^6^/ml). The infected cells were harvested 48 h postinfection, and the GST-tagged protein was purified on glutathione (GSH)–Sepharose (GE Healthcare). *Tb*DHFR-TS was unstable on cleavage of the GST tag; therefore, all experiments were carried out using GST-tagged protein.

### Determination of enzyme concentration

*Tb*PTR1 enzyme concentration was determined spectrophotometrically (extinction coefficient at 260 nm = 14,815 M^−1^ cm^−1^). Because tightly bound folate can interfere with absorbance at 260 and 280 nm, the concentration of *Tb*DHFR-TS was determined using Coomassie blue reagent [17].

The concentration of catalytically active enzyme in each preparation was determined using a titration of enzyme in the presence of excess inhibitor, methotrexate (MTX), to calculate the concentration of active sites in each sample. This concentration was used for all subsequent work.

### Novel screening assay protocol: Assay conditions

*Tb*PTR1 activity was assayed in a buffer containing 20 mM sodium citrate and 1 mM ethylenediaminetetraacetic acid (EDTA) (pH 6.0). The final reaction mixture contained test compound at a range of concentrations: *Tb*PTR1 (4.8 nM), H_2_B (0.35 μM), cyt *c* (81 μM), and NADPH (100 μM). The final assay volume was 200 μl in 96-well clear polystyrene plates with a final dimethyl sulfoxide (DMSO) level of 1% in all samples, including controls.

*Tb*DHFR-TS activity was assayed in a buffer containing 20 mM sodium citrate and 1 mM EDTA (pH 7.4). The final reaction mixture contained test compound at a range of concentrations: *Tb*DHFR-TS (1 nM), H_2_F (4.4 μM), cyt *c* (81 μM), and NADPH (100 μM). The final assay volume was 200 μl in 96-well clear polystyrene plates with a final DMSO level of 1% in all samples, including controls.

All reagents were solubilized in assay buffer with the exception of H_2_B, which was solubilized in 0.2 M NaOH. The final assay concentration of NaOH was 70 μM.

### Novel screening assay protocol: Liquid handling

Test and standard compounds were cherry-picked into the first column of a 96-well polypropylene plate and then serially diluted in 100% DMSO through 10 half-log increments in row orientation using a JANUS eight-channel Varispan automated workstation (PerkinElmer). This produced working stock (100× final concentration in assay) compound plates with six test compound curves and two standard compound curves occupying columns 1 to 10. Aliquots (2 μl) of each compound working stock were then stamped into replicate clear 96-well polystyrene assay plates using a PlateMate 2 × 2 pipetting workstation (Thermo Fisher).

Then 20 μl of cyt *c* (stock concentration of 810 μM in assay buffer) was added to all wells using the JANUS MDT I200 96-well head (PerkinElmer). Assay buffer (176 μl) containing enzyme and substrate (stock concentrations of 5.5 nM and 0.4 μM for *Tb*PTR1 and 1.12 nM and 5 μM for *Tb*DHFR-TS, respectively) was then added to columns 1 to 11 only using the same instrument. Buffer containing substrate only was added to column 12 to provide the no-enzyme control. In all liquid handling steps for reagent addition, tips were washed between each transfer and changed after two transfers.

The reaction was started by the addition of 2 μl of NADPH (stock concentration of 100 μM) using a FlexDrop reagent dispenser (PerkinElmer). Each plate was started at 1-min intervals in accordance with the time taken to read a plate. The *Tb*PTR1 assay was run for 50 min, the *Tb*DHFR assay was run for 40 min, and the absorbance was read at 550 nm using a Victor^3^ multilabel plate reader (PerkinElmer).

### Spectrophotometric assay for *Tb*PTR1

Verification of catalytic activity of *Tb*PTR1 was carried out as described by Dawson and coworkers [16]. Briefly, solutions containing *Tb*PTR1 (82.5 nM) and H_2_B (20 μM in 0.2 M NaOH) were buffered with 20 mM sodium citrate (pH 4.7). The reaction was executed in a 1-ml cuvette (990 μl) and initiated with 100 μM NADPH (10 μl). A decrease in absorbance was followed spectrophotometrically at 340 nm.

### HPLC assay for *Tb*PTR1

*Tb*PTR1 activity was assayed by directly measuring the formation of H_4_B from H_2_B at 22 °C using a previously described HPLC-based method [18] with modifications. Assays were carried out in 20 mM sodium citrate (pH 6.0) (for direct comparison with the screening assay) containing 1 mM EDTA and 50 μM NADPH cofactor. Aliquots (100 μl) of enzymatic reactions were made alkaline by the addition of 46 mM NaOH and oxidized in the presence of 12 mM KI/4.4 mM I_2_ in the dark at 22 °C for 1 h. Under these conditions, H_2_B and H_4_B were oxidized to biopterin and pterin, respectively [18,19]. Excess iodine was removed by the addition of 12 mM ascorbate. Neopterin (25 nM) was added as internal standard. Samples were then acidified with 83 mM HCl, and precipitated proteins were removed by centrifugation (16,000*g,* 10 min, 22 °C). Supernatants were analyzed by reverse-phase HPLC on an ion-paired Ultrasphere C_18_ column using a Dionex UltiMate 3000 system coupled to a Dionex RF-2000 fluorometer. The mobile phase contained 20 mM sodium phosphate and 4 to 10% methanol with a flow rate of 1 ml min^−1^. Pterins were detected fluorometrically using excitation and emission wavelengths of 360 and 440 nm, respectively. Products were quantified against pterin standards whose concentrations were determined using published extinction coefficients [20]. The linearity of the assay was established by measuring H_4_B formation over time (15–180 s) in the presence of 1.1 nM *Tb*PTR1 using 10 and 500 nM H_2_B. The enzyme proportionality of the assay was investigated by measuring H_4_B produced in 1 min by varying concentrations of *Tb*PTR1 (0.31–10 nM) using constant substrate (25 nM H_2_B). The linearity of the data sets was analyzed by linear regression. The *K*_m_ for H_2_B was determined by measuring H_4_B produced in 1 min by 1.1 nM *Tb*PTR1 using varying concentrations of H_2_B (3–300 nM). The IC_50_ for MTX was determined by measuring H_4_B produced in 1 min by 1.1 nM *Tb*PTR1 at [*S*] = *K*_m_ in the presence of varying concentrations of MTX.

### Crystallography

In preparation for crystallization, *Tb*PTR1 was concentrated to 6 mg ml^−1^ in 20 mM Tris–HCl (pH 8.0). The ligand DDD00066641 was solubilized to a concentration of 200 mM in DMSO. The ternary complex of *Tb*PTR1 with cofactor and ligand was prepared by incubating the protein solution (6 mg ml^−1^) with 2 mM of the ligand, 1 mM NADP^+^, and 20 mM dithiothreitol in 20 mM Tris–HCl (pH 8.0) at 4 °C for 30 min prior to crystallization.

Crystallization was carried out by the vapor diffusion method by mixing 2 μl of the protein solution with 2 μl of the reservoir solution and incubating the drops over 100 μl of the reservoir in sitting drop plates. The reservoir solution consisted of 1.5–3.0 M Na acetate and 0.1 M citrate buffer (pH 4.5–6.0). Diffraction quality crystals were obtained after incubation for 2 to 3 days at 18 °C.

Diffraction data were collected using a rotating anode X-ray source (Rigaku Micromax 007) and an image plate detector (Rigaku R-AXIS IV^++^). Crystals were prepared for data collection by transferring them through a cryoprotection solution of mother liquor plus 20% glycerol and then were flash frozen in a stream of gaseous nitrogen at 100 K. Data were integrated and scaled using MOSFLM [21] and SCALA [22] from the CCP4 [23] suite of programs.

Molecular replacement, as implemented in MOLREP [24], was used to solve the structure using the protein chains from the *Tb*PTR1/MTX complex [16] (Protein Data Bank [PDB] 2C7V) as the starting model. After the molecular replacement step, a round of rigid body refinement was carried out using REFMAC5 [25]. Ligand models and associated topology files were created with PRODRG [26] and were built into F_o_ − F_c_ electron density maps using COOT [27]. Further rounds of restrained refinement were carried out using REFMAC5 and manual alteration of the models, including the addition of solvent molecules using COOT. Coordinates and structure factors for *Tb*PTR1 plus DDD000066641 have been deposited in the PDB with accession code 2VZ0.

### Data analysis

For determination of *K*_m_, data were fitted by nonlinear regression in GraFit to the following general equation for substrate inhibition:

(1) $$\nu =\frac{V_{\text{max}}}{1+\frac{K_{\text{m}}}{S}+\frac{S}{K_{\text{i}}^{\text{S}}}}\text{.}$$

For determination of IC_50_ for inhibitors, all routine curve fitting was undertaken using a four-parameter logistic dose–response curve (model 205) in XLFit 4.2.

For MTX potency determination, data were fitted in GraFit to Morrison’s quadratic equation for tight-binding inhibitors [28]:

(2) $$\frac{\nu_{\text{i}}}{\nu_{0}}=1-\frac{\left({[E]}_{\text{T}}+{[I]}_{\text{T}}+K_{\text{i}}^{\text{app}}\right)-\sqrt{{\left({[E]}_{\text{T}}+{[I]}_{\text{T}}+K_{\text{i}}^{\text{app}}\right)}^{2}-4{[E]}_{\text{T}}{[I]}_{\text{T}}}}{2{[E]}_{\text{T}}}\text{,}$$

where *v*_i_ and *v*_0_ are the rates with and without inhibitor, [*E*]_T_ is the total enzyme concentration (a fixed parameter), [*I*]_T_ is the total inhibitor concentration, and *K*_i_^app^ is the apparent inhibitor constant.

For competitive inhibitors,

(3) $$K_{\text{i}}^{\text{app}}=K_{\text{i}}\left(1+\frac{\left[S\right]}{K_{\text{m}}}\right)\text{.}$$

For routine assessment of potency for tight-binding inhibitors identified through test compounds, *BatchK_i_* software (BioKin) was used to determine *K*_i_^app^. A modified Morrison equation [29] was used as the fitting model in which *v*_b_ is the background enzymatic rate, *v*_0_ is the “full” catalytic signal in the absence of inhibitor, [*E*]_0_ is the active enzyme concentration, and [*I*]_T_ is the inhibitor concentration:

(4) $$\nu =\nu_{\text{b}}+\nu_{0}\frac{{[E]}_{0}-{[I]}_{\text{T}}-K_{\text{i}}^{\text{app}}+\sqrt{{\left({[E]}_{0}-{[I]}_{\text{T}}-K_{\text{i}}^{\text{app}}\right)}^{2}-4{[E]}_{0}K_{\text{i}}^{\text{app}}}}{2{[E]}_{0}}\text{.}$$

The *BatchK_i_* software automatically determined initial estimates of *K*_i_^app^ based on a weighted average algorithm [30]. First-round analysis treated the active enzyme concentration as a fixed constant and treated *v*_b_, *v*_0_, and *K*_i_^app^ as adjustable parameters. For tight-binding inhibitors, a second round of analysis allowed determination of the enzyme active site concentration, where [*E*]_0_ is treated as an adjustable parameter [31]. The optimization of [*E*]_0_ was performed within preset limits (±5∗[*E*]_0_). The data-fitting algorithm used in *BatchK_i_* software was either Reich’s modification [32] of the Levenberg–Marquardt method [33] or a robust regression algorithm [34] based on Huber’s minimax method [35].

The *Z*′ factor for control data on each screening plate was calculated as defined by Zhang and coworkers [36], where μ_HI_ and μ_LO_ are the means and σ_HI_ and σ_LO_ are the standard deviations for the positive and negative control wells, respectively:

(5) $$Z^{\prime }=1-\frac{3(\sigma_{\text{HI}}+\sigma_{\text{LO}})}{\mu_{\text{HI}}-\mu \text{LO}}\text{.}$$

## Results

### Reduction of cyt *c* Fe^3+^ as a read-out of PTR1 activity in vitro

**Fig. 1:**
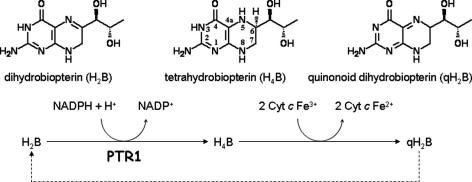
Principle of the cyt *c*-coupled assay for *Tb*PTR1. H_2_B is reduced by NADPH (ε_340nm_ = 6.22 mM^−1^ cm^−1^) and *Tb*PTR1 to H_4_B, which then nonenzymatically reduces cyt *c* Fe^3+^ to cyt *c* Fe^2+^ (Δε_550nm_ = 21.1 mM^−1^ cm^−1^) and qH_2_B [20]. Under anaerobic conditions, the stoichiometry is 2 mol cytochrome *c*/mol NADPH and 1.5- to 1.8-fold under aerobic conditions [19], leading to an increase in absorbance signal of 5- to 6-fold. qH_2_B is unstable at near neutral pH and spontaneously isomerizes principally to H_2_B [20].

Previously, Hasegawa and coworkers [37] demonstrated that H_4_B could be oxidized by ferricytochrome *c* (cyt *c* Fe^3+^) via a trihydrobiopterin radical intermediate to form quinonoid H_2_B (qH_2_B) and cyt *c* Fe^2+^ (Fig. 1 ). The formation of reduced cyt *c* Fe^2+^ can be monitored as an increase in absorbance at 550 nm. Therefore, we investigated whether this reaction could be adopted to enable detection of the product of the PTR1 and DHFR-TS enzyme reactions.

**Fig. 2:**
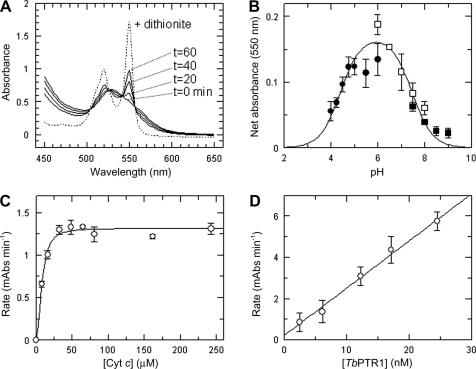
Optimization of the cyt *c*-coupled assay for *Tb*PTR1. (A) Reduction of cyt *c* by *Tb*PTR1 activity. The absorbance spectrum of cyt *c* (80 μM) was measured at intervals (20, 40, and 60 min) in the presence of *Tb*PTR1 (20 nM), H_2_B (0.5 μM), and NADPH (100 μM) using a cuvette-based assay. Controls omitting one of the components give identical spectra to the zero time trace (not shown). Na^+^ dithionite was added to completely reduce residual cyt *c.* (B) Determination of optimum pH. Assays contained 4.8 nM *Tb*PTR1, 0.35 μM H_2_B, 80 μM cyt *c*, and 100 μM NADPH in a range of buffers—Na^+^ citrate (closed circles), Na^+^ phosphate (open squares), and Tris (closed squares) (all at 20 mM)—containing 1 mM EDTA adjusted to different pH values with citric acid, phosphoric acid, and Na^+^ hydroxide, respectively. Absorbance at 550 nm was measured after 60 min. (C) Determination of optimum cyt *c* concentration. Assays contained 4.8 nM *Tb*PTR1, 0.35 μM H_2_B, and 100 μM NADPH in 20 mM Na^+^ citrate/1 mM EDTA buffer (pH 6.0) with varying cyt *c* concentrations. Absorbance at 550 nm was measured after 60 min. (D) Proportionality of activity with enzyme concentration. Assays contained varying concentrations of *Tb*PTR1, 0.35 μM H_2_B, 80 μM cyt *c,* and 100 μM NADPH in 20 mM Na^+^ citrate/1 mM EDTA buffer (pH 6.0). Absorbance at 550 nm was measured after 60 min. mAbs, monoclonal antibodies. Each data point shown is the mean ± standard deviation of triplicate determinations.

We first determined “proof of concept” for the coupling assay using a standard ultraviolet–visible (UV–Vis) spectrophotometer (Fig. 2 A). The absorbance profile of cyt *c* Fe^3+^ and cyt *c* Fe^2+^ was determined. Cyt *c* Fe^2+^ produced by an excess of solid sodium dithionite was confirmed to be a narrow defined peak at 550 nm, accompanied by the formation of an additional shallower and broader peak at 520 nm. A similar spectrum was obtained on reduction with H_4_B but not with H_2_B (not shown). However, H_2_B was able to reduce cyt *c* Fe^3+^ in the presence of *Tb*PTR1 and NADPH in a time-dependent manner (Fig. 2A).

### Development of a microtiter plate-based screening assay

From this point forward, all work was carried out in a 96-well plate format with a Victor^3^ multilabel plate reader (PerkinElmer) using a 550 ± 9-nm filter. The optimum pH for the coupled assay was determined to be pH 6.0 in either citrate or phosphate buffer (Fig. 2B). Cyt *c* concentrations greater than 50 μM gave maximal rates of reduction (Fig. 2C), and rates of cyt *c* reduction are proportional to enzyme activity up to 25 nM (Fig. 2D).

**Fig. 3:**
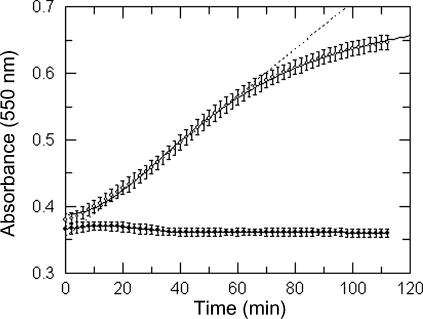
Time-based linearity of cyt *c*-coupled assay for *Tb*PTR1. Using final assay conditions described in Materials and Methods, signal generation was measured across a time course. Open symbols: plus enzyme (4.8 nM); closed symbols: minus enzyme. The dotted line is the linear regression of the change in absorbance at 550 nm from 20 to 50 min. Data shown are from a representative experiment that was reproduced on three independent occasions. Each data point shown is the mean ± standard deviation of 16 replicates.

Following a lag period, reduction of cyt *c* was linear from 20 to 60 min (*r* ^2^ = 0.9994) and, therefore, was suitable for kinetic analyses (Fig. 3 ). The increase in *A*_550nm_ over this period in the absence of *Tb*PTR1 was negligible and indicates that NADPH itself does not significantly reduce cyt *c.* For routine inhibitor screening, plates were read after 50 min to ensure that a maximal signal was achieved. At this time point, 23.9 μM of product (cyt *c* Fe^2+^) had been formed from 0.35 μM H_2_B in the reaction mixture. Based on the stoichiometry of 1.5 to 1.8 for reduction of cyt *c* by H_4_B under aerobic conditions, H_2_B had been cycled 40–46 times after 50 min, and measuring linear rates of reaction between 20 and 50 min, the signal-to-background ratio for the assay was determined to be 43. Given that not more than 10% of H_2_B can be used in the direct assay, when measuring the initial linear rate of absorbance change at 340 nm due to the oxidation of NADPH, it is evident that the coupled assay is approximately 400-fold more sensitive than the direct assay method.

Termination of the reaction can be achieved with 10 μl of NaOH (0.2 mM), although use of a rapid read plate reader dispensed with the need for this additional step in our studies. Initial performance statistics of the microtiter plate-based assay demonstrated its clear potential for routine compound assessment purposes, yielding highly acceptable *Z*′ values (Table 1 ).

### Determination of kinetic parameters

**Fig. 4:**
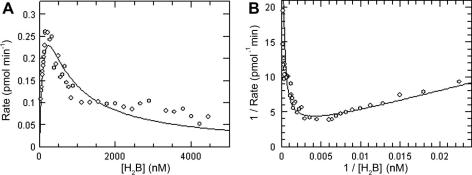
Determination of *K*_m_^app^ for H_2_B using the coupled assay. (A) Enzyme rate versus substrate concentration. The curve represents the best nonlinear fit to the general equation for substrate inhibition. (B) Lineweaver–Burk transformation of data. The resulting kinetic parameters were as follows: *K*_m_ = 167 ± 54 nM, *K*_i_^s^ = 439 ± 135 nM, *V*_max_ = 0.51 ± 0.10 pmol min^−1^, and *k*_cat_/*K*_m_ = 5.6 × 10^5^ M^−1^ s^−1^. Data shown are from a representative experiment that was reproduced on three independent occasions. Each data point shown is the mean of duplicate determinations.

Using the coupled assay, the linear rate of product formation was measured over a wide range of H_2_B concentrations. The resulting plot of rate versus substrate concentration revealed pronounced inhibition by H_2_B (Fig. 4 ), as reported previously for the *T. brucei* [16] and *L. major* enzymes [11]. Fitting the data to the standard equation for substrate inhibition (Eq. (1)) gave an apparent *K*_m_ (*K*_m_^app^) of 151 ± 50 nM, a substrate inhibition constant (*K*_i_^s^) of 439 ± 135 nM, and a *k*_cat_ of 0.0016 ± 0.0003 s^−1^. These kinetic parameters are considerably lower than those reported previously [16] but were determined at pH 6.0 (optimal in this assay format) rather than at the previously reported pH optimum of 3.7.

**Fig. 5:**
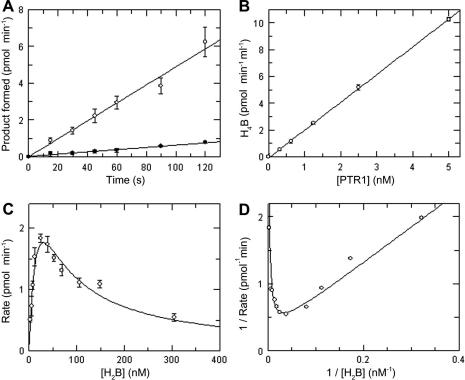
*Tb*PTR1 assay by HPLC. All assays were carried out in 20 mM citrate, 1 mM EDTA, and 50 μM NADPH (pH 6.0) with specified concentrations of H_2_B and initiated with *Tb*PTR1. Formation of H_4_B product was determined by HPLC as described in Materials and Methods. (A) Linearity of assay at high and low substrate concentrations. Assays contained 1.1 nM *Tb*PTR1, and reactions were terminated at the specified time intervals. Open circles: 10 nM; closed circles: 500 nM H_2_B. (B) Proportionality of activity with enzyme concentration. Assays contained 25 nM H_2_B, and reactions were terminated after 1 min. (C) Enzyme rate versus substrate concentration. Assays contained 1.1 nM *Tb*PTR1, and reactions were terminated after 1 min. The curve represents the best nonlinear fit to the high substrate inhibition equation. (D) Lineweaver–Burk transformation of kinetic data. The resulting kinetic parameters were as follows: *K*_m_ = 25.0 ± 6.7 nM, *K*_i_^s^ = 37.2 ± 10.4 nM, *V*_max_ = 4.7 ± 0.9 pmol min^−1^, and *k*_cat_/*K*_m_ = 2.8 × 10^6^ M^−1^ s^−1^. Data points represent the means and standard deviations of triplicate measurements. Data shown are from representative experiments that were reproduced on three independent occasions. Each data point shown is the mean of triplicate determinations.

To confirm the validity of the coupled assay, kinetic parameters with respect to substrate were also determined using an alternative HPLC-based assay for PTR1 activity determination (Fig. 5 ). Using this method, formation of H_4_B product was stoichiometric with the disappearance of H_2_B, linear for at least 2 min across the assay range (Fig. 5A), and rates determined after 1 min of incubation were proportional to enzyme up to at least 5 nM *Tb*PTR1 (*r* ^2^ = 0.9999) (Fig. 5B). As noted previously, the enzyme is inhibited at high substrate concentrations, yielding kinetic parameters *K*_m_^app^ = 25 ± 6.7 nM, *K*_i_^s^ = 37.2 ± 10.4 nM, and *k*_cat_ = 0.070 ± 0.013 s^−1^ (Fig. 5C and D). The 7-fold discrepancy in *K*_m_^app^ between the coupled recycling assay and the HPLC assay most likely reflects the fact that, under steady-state conditions in the coupled assay, the biopterin substrate is present as a mixture of H_2_B, H_4_B, and qH_2_B (see Fig. 1).

For standard inhibitor screening, it is ideal to run an assay at or below *K*_m_ for competing substrate to maximize the chances of identifying activity. However with significant substrate inhibition and limited signal-to-background ratio in this assay, an H_2_B concentration giving an optimal window of signal in the coupled assay was selected for routine screening. The second substrate, NADPH, was saturating in both assays given that the *K*_m_^app^ is 1.6 ± 0.1 μM, as determined by the HPLC assay method (data not shown).

Based on these studies, the final optimized assay conditions used for compound screening were defined as 0.3 μM H_2_B, 100 μM NADPH, 81 μM cyt *c* Fe^3+^, and 4.8 nM *Tb*PTR1 in 20 mM sodium citrate buffer plus 1 mM EDTA (pH 6.0). These conditions were expected to be conducive to the identification of inhibitors binding at the substrate site of *Tb*PTR1.

### Inhibition by MTX

**Fig. 6:**
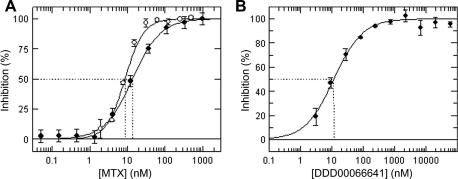
Inhibition of *Tb*PTR1 by MTX and DDD00066641. (A) Concentration-dependent inhibition by MTX. MTX potency was determined using the cyt *c*-coupled assay (open circles, *n* = 16) and the HPLC assay (closed circles, *n* = 3). For the coupled assay, the mean IC_50_ was 13.7 ± 2.1 nM and the Hill slope was 1.19 ± 0.14. The corresponding *K*_i_^app^ was 11.1 ± 2.1 nM. For the HPLC assay, the corresponding values were IC_50_ = 8.22 ± 0.21 nM, Hill slope = 2.1 ± 0.1, and *K*_i_^app^ = 7.1 ± 1.0 nM. (B) Concentration-dependent inhibition by DDD00066641 as determined using the cyt *c* method. The IC_50_ value was 10.6 nM, the Hill slope was 0.98, and the corresponding *K*_i_^app^ was 9.8 ± 2.6 nM.

Further validation of the coupled assay was obtained through the use of the established antifolate MTX as a standard inhibitor. An average IC_50_ of 13.7 ± 2.1 nM (*n* = 16) and a Hill slope of 1.19 ± 0.14 (*n* = 16) were determined using the newly developed screening assay (Fig. 6 A). Given the enzyme concentration (4.8 nM), MTX potency is within tight-binding territory [38]. Therefore, *K*_i_^app^ analysis was undertaken to report potencies more accurately (Eq. (2)). The corresponding *K*_i_^app^ was 11.1 ± 2.1 nM. MTX potency against *Tb*PTR1 was validated using the HPLC assay, yielding an IC_50_ of 8.2 ± 0.2 nM and a *K*_i_^app^ of 7.1 ± 1.0 nM. Based on the *K*_m_^app^ and H_2_B concentrations used in the respective assays (Eq. (3)), the *K*_i_ values for MTX were in excellent agreement (4.1 and 3.6 nM for the coupled and HPLC assays, respectively).

Furthermore, Fig. 6B shows a typical concentration–effect curve in the screening assay for a compound representing one of the novel chemical series identified through our medicinal chemistry efforts.

### Assay development for *Tb*DHFR-TS

To produce an effective therapy for HAT, it is possible that inhibition of both *Tb*PTR1 and *Tb*DHFR-TS will be required. Therefore, the application of the coupled assay to measure DHFR activity in vitro was investigated. The coupled assay format was found to support the measurement of both human and *Tb*DHFR-TS activity, in particular using a sodium citrate buffer (pH 7.4) to optimize the window of signal achievable.

**Fig. 7:**
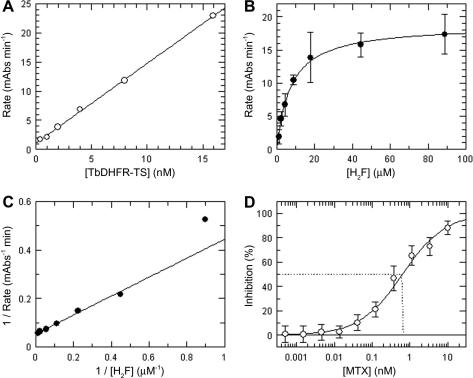
Summary of *Tb*DHFR-TS assay development for the cyt *c*-coupled assay method. (A) Proportionality of *Tb*DHFR-TS activity with enzyme concentration. (B) Enzyme rate versus substrate concentration. (C) Lineweaver–Burk transformation of data. (D) IC_50_ for MTX. The average MTX IC_50_ (*n* = 16) against *Tb*DHFR-TS was 0.64 nM ± 0.28 nM, and the Hill slope was 0.78 ± 0.08. The corresponding *K*_i_^app^ was 0.24 ± 0.14 nM. mAbs, monoclonal antibodies. Data shown are from representative experiments that were reproduced on three independent occasions. Each data point shown is the mean of duplicate determinations from two independent experiments.

Under these conditions, activity was proportional up to at least 15 nM *Tb*DHFR-TS (Fig. 7 A) and the assay was linear between approximately 2 and 55 min. The enzyme displayed normal Michaelis–Menten kinetics, with H_2_F as substrate yielding a *K*_m_^app^ of 7.3 ± 1.5 μM (Fig. 7B and C). MTX was shown to be a very potent inhibitor of *Tb*DHFR-TS, with an average IC_50_ of 0.64 nM ± 0.28 nM and an average Hill slope of 0.78 ± 0.08 (*n* = 16) (Fig. 7D). Because MTX proved to be a tight-binding inhibitor of *Tb*DHFR-TS, the corresponding *K*_i_^app^ was calculated for MTX to be 0.24 ± 0.14 nM, yielding a *K*_i_ of 0.15 nM.

The assay performance statistics generated from assay development and mock screening studies for the DHFR assay are shown in Table 1. As for the PTR1 assay, they yielded readily acceptable *Z*′ values.

### Lead generation for *Tb*DHFR-TS and *Tb*PTR1

**Fig. 8:**
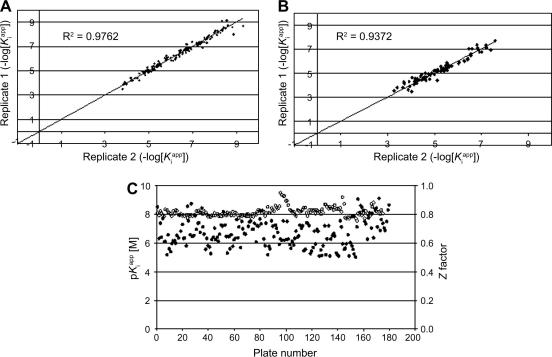
Longitudinal performance monitoring of the novel screening assay. Concentration-dependent inhibition was determined for all compounds screened against *Tb*PTR1 and *Tb*DHFR-TS. Briefly, serial dilution curves (10-point curves, 1:3 dilutions in DMSO) were created and assayed in duplicate using the cyt *c*-coupled assay. All assays were performed as described in Materials and Methods. The correlation between replicate p*K*_i_^app^ (−log *K*_i_^app^) values is shown for *Tb*PTR1 (A) (*n* = 137 compounds) and *Tb*DHFR-TS (B) (*n* = 80 compounds). (C) Performance of the cyt *c*-coupled assay across a period of 18 months. Closed circles: *Z*′ values; open circles: MTX p*K*_i_^app^ values from all assay plates.

The program was initiated using two approaches: ligand-based design and in silico screening. The identification and optimization of PTR1 and DHFR-TS inhibitory activity were underpinned by data provided by the newly developed screening assay and structure-guided chemistry design. Throughout the project, potency values (*K*_i_^app^ from 10-point concentration–effect curves, *n* = 2) were generated for *Tb*PTR1, *Tb*DHFR-TS, and (for selectivity purposes) human DHFR (Eq. (4)). Fig. 8 illustrates the high level of correlation observed between replicate potency values for a sample of test compounds in both PTR1 and DHFR-TS assays. These data provided early confidence that the assay developed could robustly support the development of structure–activity relationships for the compound series investigated. Fig. 8C illustrates the highly consistent performance of the PTR1 assay with respect to the key metrics of *Z*′ and MTX potency over a time period spanning 18 months. The ultimate validation of this novel screening assay was confirmed on demonstration that compounds identified as actives using the new assay were in fact legitimately binding to *Tb*PTR1.

**Fig. 9:**
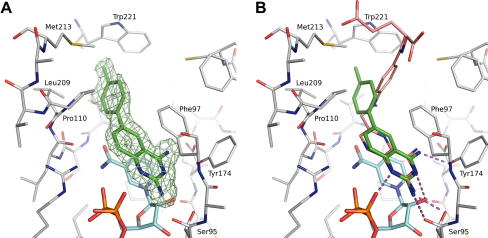
Cocrystallization of key compounds with *Tb*PTR1: Detailed views of the *Tb*PTR1 active site in complex with NADPH and DDD00066641. (A) Binding mode of DDD00066641 (green carbon atoms) and the cofactor NADPH (cyan carbon atoms) in the active site (gray carbon atoms). Omit electron density, calculated after one round of refinement with the ligand atoms removed from the model, is shown as green mesh, contoured at 3 sigma. Key residues are labeled for clarity. (B) Binding mode of DDD00066641 (green carbon atoms) superimposed on the binding mode of MTX (pink C atoms). Hydrogen bonds between the ligand and the protein/cofactor are shown as dashed lines, and key residues are labeled for clarity. (For interpretation of the references to color in this figure legend, the reader is referred to the Web version of this article.)

This is exemplified using compound DDD00066641 (6-*p*-tolylquinazoline-2,4-diamine), which was identified as a potent inhibitor of *Tb*PTR1 using the coupled assay, with an average *K*_i_^app^ of 9.8 ± 2.6 nM (Fig. 6B). For a competitive inhibitor, *K*_i_ = *K*_i_^app^ (1 + *S*/*K*_m_), yielding a *K*_i_ of 3.0 ± 0.8 nM. The structure of the ternary complex of *Tb*PTR1 with this ligand and the cofactor NADP^+^ was obtained by cocrystallization (Fig. 9 A). The binding mode of the ligand is similar to that observed for MTX bound to *Tb*PTR1 [16], with the diaminoquinazoline ring of DDD00066641 sandwiched between the nicotinamide ring of the cofactor and the side chain of Phe97 (Fig. 9B). The diaminoquinazoline ring of DDD00066641 also shows an identical pattern of H-bond interactions to the protein chain and cofactor, as observed for the pteridine ring of MTX. The ligand forms three H-bonds to the protein: the 2 amino group interacting with both the main chain carbonyl and side chain hydroxyl of Ser95 and the 4 amino group interacting with the side chain hydroxyl of Tyr174. Two H-bonds are also formed via the N1 atom to the α-phosphate group and from the N3 atom to the nicotinamide ribose 2′ hydroxyl. The hydrophobic toluene substituent packs against a hydrophobic surface formed by the side chains of Leu209, Pro210, Met213, and Tyr221.

The medicinal chemistry program to date has yielded a number of compound series with single digit nanomolar *K*_i_^app^ values for *Tb*PTR1. Furthermore, individual series have been developed with equipotent, *Tb*PTR1-selective, and *Tb*DHFR-TS-selective profiles providing the potential to comprehensively probe the biology of pterin and folate metabolism in the African trypanosome. Routine cocrystallization studies with *Tb*PTR1 across these series have repeatedly confirmed the binding mode revealed for DDD00066641.

## Discussion

In this article, we have presented the development and validation of a nonenzymatically coupled microtiter plate-based assay for PTR1 and DHFR-TS. The assay was developed to enable a drug discovery project to identify and develop small-molecule inhibitor series of both PTR1 and DHFR-TS in the African trypanosome. The assay platform described in this article was thoroughly validated as an accurate read-out of PTR1 and DHFR-TS activity in vitro through a number of experimental approaches.

The potency of a known inhibitor, MTX, observed against *Tb*PTR1 using the novel screening assay (IC_50_ = 13.7 ± 2.1 nM) was in good agreement with the potencies reported for *L. major* [10,11,39], *Leishmania tarentolae* [39], and *Trypanosoma cruzi* [40]. Similarly, the IC_50_ of MTX observed against *Tb*DHFR-TS using the coupled assay (IC_50_ = 0.64 ± 0.28 nM and *K*_i_ = 0.15 nM) was in agreement with the IC_50_ values reported for human, *Escherichia coli,* and *Pneumocystis* *carinii* DHFR-TS enzymes [41]. Further validation was provided by comparison of MTX potency in an orthogonal platform for *Tb*PTR1. The HPLC assay for PTR1 yielded an IC_50_ of 8.2 ± 0.2 nM, again in excellent agreement with that produced by our screening assay. Calculation of *K*_i_ values taking into account tight-binding behavior and substrate load of the respective assays produced nearly identical values (4.0 and 3.5 nM for screening and HPLC assays, respectively).

Analysis and comparison of PTR1 and DHFR-TS substrate *K*_m_ values between assay platforms revealed some interesting observations. Whereas DHFR-TS enzymes are restricted in their substrate repertoire, primarily reducing H_2_F to H_4_F, PTR1 can reduce numerous pterins and folates. Furthermore, there are substantial interspecies differences in PTR1 activity and substrate sensitivities [42]. Substrate inhibition of *Tb*PTR1 in response to H_2_B has also been reported in both *Tb*PTR1 and *Lm*PTR1 [16] but has not been reported for DHFR-TS in any species. Consistent with the literature, substrate titrations at a fixed enzyme concentration identified substrate inhibition of *Tb*PTR1 (with H_2_B) but not of *Tb*DHFR-TS. The *K*_m_^app^ for *Tb*DHFR-TS determined in the screening assay agrees well with literature reports [43–45].

The *K*_m_^app^ for *Tb*PTR1 in the screening assay was determined to be 167 ± 54 nM, which is considerably lower than the previously reported *K*_m_ for *Tb*PTR1 (10.9 ± 2.4 μM) [16]. However, the *K*_m_^app^ was determined at pH 6.0 in the current study, not at pH 3.7 as described by Dawson and coworkers [16]. To support the determination of *K*_m_ using the screening assay, *K*_m_ was also determined using the HPLC assay at pH 6.0. Substrate inhibition with respect to H_2_B was also observed in the HPLC assay, and a nanomolar *K*_m_^app^ was determined (25 ± 6.7 nM). This small shift in apparent affinity for substrate between the screening assay and the HPLC assay is most likely a result of the mixture of biopterin species present in the coupled assay (H_2_B and qH_2_B) that are not present at steady-state levels in the HPLC assay. It is also possible that the mixture of species in the coupled assay contributes significantly to the initial lag phase observed, with qH_2_B possibly being a more efficient substrate for PTR1 than H_2_B itself and linearity being achieved only after a steady state of these species has been attained. In support of this notion, the lag phase is considerably shorter in the equivalent DHFR assay.

The ultimate validation of the ability of a screening assay to robustly identify inhibitors is in the confirmation of identified actives as genuine binders to the target in question. We have shown, using cocrystallization studies, that an example small molecule identified using the screening assay described here binds to *Tb*PTR1 through interactions within the pterin binding pocket in a manner similar to that observed for MTX. Furthermore, the structure–activity relationships developed and informed by data generated by the screening assay conform well to the binding modes identified for multiple series through similar cocrystallization studies.

Another key attribute for a screening assay is that it should be deemed as fit for purpose. This screening assay was developed to enable a structure-guided medicinal chemistry project and, therefore, was required to produce accurate reproducible (*n* = 2) potency data for up to three targets (*Tb*PTR1, *Tb*DHFR-TS, and human DHFR) for up to 20 compounds each week for a period of at least 12 months. We have demonstrated in this article that the platform developed was able to consistently produce assay statistics that were highly acceptable in terms of individual plate *Z*′ values and returned potency values for the known inhibitor MTX that were within expectations based on comparison with literature and alternative assay platforms. Furthermore, these key performance metrics were maintained longitudinally over the time period of the project, allowing high levels of plate approval and minimal rescreening. Across a period of 18 months, the project produced approximately 1500 approved concentration–effect curves employing the novel screening assay.

In summary, we have developed a screening assay that is suitable for routine inhibitor assessment for both PTR1 and DHFR-TS targets. The assay principle relies on H_4_B and H_4_F, the products of catalysis by *Tb*PTR1 and *Tb*DHFR-TS, respectively, reducing cyt *c* and resulting in an increase in signal at 550 nm. Unlike the conventional spectrophotometric absorbance assay measuring oxidation of NADPH at 340 nm, the novel assay provides the throughput, enhanced sensitivity, and extended period of linearity required of an end-point drug discovery screening assay. We believe that the enhanced sensitivity and extended window of linearity are the result of a cycling reaction involving H_4_B, which itself reduces cyt *c* forming qH_2_B and which can subsequently dissociate to H_2_B. The HPLC assay also described in this article will be used to support detailed mode of inhibition studies for key compounds. Full details of enzymatic and biological activities of inhibitors discovered using this assay method will be published separately [46].

## Figures and Tables

**Table 1:** Statistical analysis of plate data.

	*Tb*PTR1	*Tb*DHFR-TS
High signal (absorbance units)	0.44 ± 0.02	0.43 ± 0.02
Low signal (absorbance units)	0.32 ± 0.01	0.33 ± 0.01
Signal/Background ratio (>1.2)^a^	1.39 ± 0.04	1.30 ± 0.03
*Z*′ (>0.5)^b^	0.77 ± 0.08	0.67 ± 0.08

*Note.* Shown are assay performance statistics of *Tb*PTR1 and *Tb*DHFR-TS in the cyt *c*-coupled assay. The assay was carried out in 96-well plates using sodium citrate/EDTA buffer at pH 6.0 and pH 7.4 for *Tb*PTR1 and *Tb*DHFR-TS, respectively. Data shown are means ± standard deviations from 26 plates of assay development and mock screening plates. Values in parentheses represent thresholds of acceptance for screening plate approval.

a When the signal/background ratio is calculated using reaction rates in the presence and absence of enzyme, a typical ratio for the assay is 43.

b Eq. (5)[36].

## Footnotes

1 *Abbreviations used:* HAT, human African trypanosomiasis; H_4_B, 5,6,7,8-tetrahydrobiopterin; H_4_F, 5,6,7,8-tetrahydrofolate; PTR1, pteridine reductase 1; NADPH, nicotinamide adenine dinucleotide phosphate; DHFR-TS, dihydrofolate reductase–thymidylate synthase; SHMT, serine hydroxymethyltransferase; dTMP, thymidylate; H_2_F, dihydrofolate; B, biopterin; H_2_B, 7,8-dihydrobiopterin; DMPK, drug metabolism and pharmacokinetics; *Tb*DHFR-TS, *T. brucei* DHFR-TS; *Tb*PTR1, *T. brucei* PTR1; HPLC, high-performance liquid chromatography; cyt *c,* cytochrome *c*; PCR, polymerase chain reaction; GST, glutathione *S*-transferase; GSH, glutathione; MTX, methotrexate; EDTA, ethylenediaminetetraacetic acid; DMSO, dimethyl sulfoxide; PTB, Protein Data Bank; cyt *c* Fe^3+^, ferricytochrome *c*; qH_2_B, quinonoid H_2_B; UV–Vis, ultraviolet–visible.
